# Brain Imaging and Impulse Control Disorders in Parkinson’s Disease

**DOI:** 10.1007/s11910-019-0980-5

**Published:** 2019-08-08

**Authors:** Andreas–Antonios Roussakis, Nicholas P. Lao–Kaim, Paola Piccini

**Affiliations:** Neurology Imaging Unit, Imperial College London – Hammersmith Hospital, 1st Floor, B–Block, Du Cane Road, London, W12 0NN UK

**Keywords:** Parkinson’s disease, Impulse control disorders, Neuroimaging, PET, SPECT, MRI

## Abstract

**Purpose of Review:**

Parkinson’s disease (PD) has a wide spectrum of symptoms including the presence of psychiatric disease. At present, most treatment plans, comprised of dopaminergic drugs, are chronic and complex. Though dopaminergic agents are quite efficient in managing the motor aspects of the disease, chronic pharmacotherapy specifically with dopamine receptor agonists has been highly linked to the occurrence of Impulse Compulsive disorder (ICD), which can be problematic for individual patients.

**Recent Findings:**

Much of what is known today about PD-related ICD stems from brain imaging studies, however, evidence is not quite conclusive. Research in the field has been focused on identifying the underlying mechanisms of PD-related ICD and understanding the functions of the structures involved in the reward network.

**Summary:**

This article presents an update of recent findings from key neuroimaging studies in PD-related ICD, discusses results from controversial studies, and identifies areas for future research in the field.

## Introduction — Definition and Overview of PD-Related ICD

Impulse Control Disorder (ICD) is a class of psychiatric disorders that present as alteration of one or more behaviours. In Parkinson’s disease (PD), ICD typically refers to compulsive gambling (or pathological gambling), hypersexuality (increased pursuit of having sex), binge-eating, excessive shopping, dopamine dysregulation syndrome (compulsivity for PD medication overuse) and punding (stereotyped, repetitive and purposeless behaviours). Within the spectrum of PD-related ICDs, hobbyism and walkabout are also described [1]. Hobbyism refers to repetitive and purposeless writing, repairing things, working on purposeless projects and excessive computer use, while walkabout refers to excessive and purposeless walking or driving. ICD severity varies greatly among PD individuals who are affected by it and can range from mild to problematic, often being intrusive with common life activities. On several occasions, certain impulsivity-driven behaviours may lead to avoidance of social interactions and disintegration of family relationships, while pathological gambling can have serious financial implications [2, 3]. The clinical management of PD-related ICD is therefore essential.

The management of PD-related ICD should be individualized. It is often challenging and most commonly comprises modification of complex dopaminergic regimes [4]. Depending on how problematic the ICD syndrome is, current guidance suggests clinical follow-up at regular intervals. A common therapeutic strategy is to reduce, replace or even discontinue certain dopaminergic drugs, most commonly dopamine receptor agonists (DAg). Clinical monitoring is thus most important to ensure that ICD improves as well as to exclude the occurrence of withdrawal symptoms. Compulsions for certain behaviours are reward-seeking, acting as a mechanism to reduce the anxiety induced by persistent thoughts. Hence, talking therapies (most commonly specialist cognitive behavioural therapy) can be quite beneficial in PD-related ICD as an adjunct to regime modification [5]. In advanced cases, deep brain stimulation has also been tried to improve ICD, however, this is not yet translated into wide scale clinical practice [6, 7].

## Evidence from Epidemiology Studies

Multiple ICDs are less common in PD (experienced by 8.8% of PD patients versus 1.3% of controls), as compared to single ICD [8]. Epidemiology studies have estimated variable prevalence rates for PD-related ICD; approximates average towards a ~ 30% prevalence among PD individuals [1, 8–10]. Though ICD is more common in PD than in the general population, the variability in prevalence rates within PD may be due to differences in study designs and evaluation methods, as well as differences in the social and cultural background of the studied populations [11].

The consensus is that higher ICD prevalence rates are related to dopamine replacement therapy (DRT) and in particular to an increased exposure to DAg [10, 12]. Interestingly, people with restless leg syndrome, who are commonly treated with DAg may be at equally high risk for developing ICD [13, 14]. PD patients who are on monotherapy with either levodopa or monoamine oxidase inhibitors have also been described to develop this behavioural disorder [15–17]. Additionally, the prevalence of ICD in drug-naïve PD patients who develop ICDs has been found at a rate similar to that seen in the general population [2]. However, as the majority of PD patients treated with dopaminergic drugs do not experience this disorder, it is difficult to extract a conclusion about the specific effects that DRT has prior to and at ICD onset, simply by reviewing its prevalence rates.

Observational epidemiology studies have identified additional risk factors for ICD including younger age at PD onset, male sex, being unmarried, having a history of smoking, depression and a family history of pathological gambling [16, 18, 19]. In fact, compulsive behaviours in the general population are commonly associated with psychiatric co-morbidities and particularly with depression [20–23]. From the above, it seems quite possible that DRT has an important role on the development/occurrence of ICD, and that early PD pathology per se may not in itself represent an increased risk. PD-related ICD may therefore have some similarities to the compulsive disorders that develop in the general population, suggesting a possible overlap in the neurochemistry of the two [24]. To understand these mechanisms better, brain imaging has provided significant evidence over the past few years. The findings of key neuroimaging studies in the field are discussed below.

## Overview of the Designs of the Imaging Studies Discussed in this Article

Most of the recent imaging studies presented here share a rather common study design: PD patients who experience an ICD, abbreviated here as PD(ICD)+, are directly compared to a group of PD patients without a history of ICD, abbreviated as PD(ICD)−. The imaging techniques employed in most published reports include several structural and functional magnetic resonance imaging (MRI) sequences and serial positron emission tomography/single photon emission computed tomography (PET/SPECT) studies. Data from matched healthy control groups are often included for comparison to increase robustness. It should be noted that most studies in the field consider PD-related ICD as one clinical entity and do not differentiate between specific compulsive behaviours. With current knowledge, the study of underlying ICD mechanisms is designed on this basis i.e. patients with one specific ICD are generally not compared against a group of patients with another ICD but to a group of PD(ICD)− patients.

Generally, the reward network is composed of dopaminergic, glutamatergic and GABAergic terminals within the cortico-basal ganglia-thalamocortical loop [25, 26]. The ventral striatum (in particular the nucleus accumbens) and its related cortical and subcortical structures therefore stand as the key regions of interest for the study of reward in this context. Indeed, as the ventral striatum is commonly implicated in ICD, drug addiction and depression in the general population, this region is of particular interest in most studies of PD-related ICD [27].

## Evidence from MRI Studies

### Functional Connectivity Studies

The ventral striatum is anatomically comprised of the nucleus accumbens, the medio-ventral part of the caudate and putamen nuclei, the olfactory tubercle and the anterior perforated substance [28, 29]. The nucleus accumbens represents the main input nucleus within the basal ganglia. In humans, the nucleus accumbens shell is connected to limbic structures and is believed to have an important role in motivation and process of emotions. The core of the nucleus accumbens is connected to the neostriatum and is related to movement [27]. Considering the above, it is worth questioning, whether PD(ICD)+ patients have pre-existing structural and functional alterations in these regions and understand the effects that exogenous dopaminergic compounds may have on them.

In comparison to PD(ICD)− patients, PD(ICD)+ patients have shown significantly lower blood oxygenation level dependent activity in the ventral striatum during risk taking and significantly reduced resting regional cerebral blood flow in the same region [30]. Ventral striatum to anterior cingulate cortex connectivity correlated with reward learning in PD(ICD)+ patients, however, not with punishment-avoidance learning [31].

At resting state, the anterior putamen appears in PD(ICD)+ patients as functionally disconnected from both anterior cingulate gyrus and inferior temporal gyrus [32]. Moreover, PD(ICD)+ patients have shown increased connectivity within the salience network (anterior insula and dorsal anterior cingulate cortex) and the central executive network (dorsolateral prefrontal and lateral posterior parietal cortex) [33]. Compared to PD(ICD)− patients and controls, patients with punding have also shown higher functional connectivity between bilateral habenula and left frontal and precentral cortices [34]. Elevated connectivity in PD(ICD)+ patients has also been observed between the ventral striatum and the limbic loop to the anterior cingulate gyrus, orbitofrontal cortex, insula, putamen, globus pallidus, and thalamus [31].

The amygdala is commonly linked to reward learning and PD(ICD)+ patients with punding have shown lower functional connectivity between the amygdala and the hippocampus [34]. Of interest, PD(ICD)+ patients have shown a strong trend for elevated amygdala-to-midbrain connectivity, only when those patients were scanned while on DAg and not while on levodopa, suggesting that DAg may indeed promote impulsivity in those patients through this route. PD(ICD)+ patients have shown decreased fMRI activation in the dorsolateral prefrontal cortex, and bilateral striatum [31, 35]. Vast functional connectivity changes have also been demonstrated in the caudate; PD(ICD)+ patients have shown decreased connectivity to the superior parietal cortex and increased connectivity to the insula, indicating that orbito-fronto-striatal and mesolimbic functional disruptions are possibly not the only mechanisms underlying PD-related ICD [35].

The studies above together suggest that in PD-related ICD, connectivity is dysfunctional within and between dopaminergic neuronal circuitries involving important subcortical and limbic-cognitive cortical regions. Among these, the default-mode, the salience and the central-executive networks seem to show consistent involvement.

### Cortical Thickness and Diffusion Tensor Imaging (DTI) Studies

Cortical thickness studies have not been greatly conclusive. PD(ICD)+ patients appear to have higher cortical thickness in the anterior cingulate cortex and rostral pole as well as in orbitofrontal cortex when compared to PD(ICD)− patients [36, 37]. Of interest, cortical thickness in the anterior cingulate and orbitofrontal cortices positively correlated with respective ICD severity scores [37]. In contrast, another study group employing cortical thickness analysis could not differentiate these three groups of subjects [32]. In a prospective study design, PD patients who later developed an ICD were indistinguishable in terms of cortical thickness to PD patients who remained free of ICD at follow-up [38]. Furthermore, there was no longitudinal difference in atrophy rate of grey matter, white matter and cortical thickness [38]. Elsewhere, PD(ICD)+ patients have shown precentral gyri and superior frontal cortical thinning as compared to PD(ICD)− patients, as well as in pars orbitalis and in inferior frontal gyrus as compared to both controls and PD(ICD)− patients [34, 39, 40]. It is likely that the corticometric changes shown in some of the above studies reflect the lack of inhibition that compulsivity is related to. However, as the above results have not been in great agreement, further projects will be needed to provide additional evidence on how important cortical thickness is in this context.

PD(ICD)+ patients as well as drug-naïve PD patients have been shown through DTI tractography to have widespread white matter tract damage in particular showing increased radial and axial diffusivity of the genu of corpus callosum and the pedunculopontine tract [39, 41, 42]. In another report using tract-based spatial statistics PD(ICD)+ patients showed increased fractional anisotropy in several white matter tracts that also included the anterior corpus callosum. However, white matter in regions known to be involved in reward-related behaviours were preserved [43]. Taken together, it seems that white matter connectivity may be indeed an important parameter to consider in designing future studies in PD-related ICD.

## Evidence from PET and SPECT Studies

### Metabolism

One of the first PET studies in ICD was focused on identifying differences between healthy controls and PD(ICD)− patients, by using ^18^F-Fluoro-2-deoxy-2-d-glucose (^18^F-FDG) PET during a gambling paradigm [44]. During the task, all healthy controls had increased metabolism in the cingulate, mesial prefrontal and fronto-orbital cortex, while PD(ICD)− did not have any frontal mesial activations, suggesting a possible impairment of the limbic loop [44]. Further to that, PD gamblers have demonstrated changes in regional cerebral blood flow (measured with ^15^O-H_2_O PET) in orbito-frontal cortex, rostral cingulate zone, amygdala and external globus pallidum while on apomorphine and during a gambling task as compared to off-apomorphine state [45]. The aforementioned change positively correlated to gambling severity, suggesting that apomorphine may reduce inhibition in dysfunctional regions of control related to impulsivity [45]. In another design, ^15^O-H_2_O PET has shown in PD patients who had no history of ICD that pramipexole increases impulsivity and that this effect is dependent on reward magnitude [46]. In that study ^15^O-H_2_O PET activation in PD(ICD)+ patients was increased in the medial prefrontal cortex and posterior cingulate cortex while it was reduced in the ventral striatum [46]. Conversely, during the Go/No-Go task (designed to assess the ability to inhibit a response), pramipexole did not have any significant effect on regional cerebral blood flow [46].

In line with the studies above, higher metabolism rates (as measured with ^18^F-FDG PET) have been shown in the orbitofrontal cortex, anterior cingulate cortex, and insula in PD patients with higher impulsivity scores [47]. PD(ICD)+ patients have shown relatively increased metabolism in the middle and inferior temporal gyri as compared to PD(ICD)− subjects; connectivity was increased mostly with both the parahippocampus and the caudate [48•].

Thus, it is likely that high impulsivity in PD is associated with increased metabolism within the fronto-insular network. Reviewing the above results, it seems that DAg may indeed affect regional function in PD patients who are susceptible to addiction. Hence, DAg-induced disruption of inhibition alongside restricted activity in executive control regions (external pallidum) may partly explain in PD the structural and functional alterations behind the control of impulse and the impaired inhibition.

### Targeting the Post-Synaptic Dopamine Receptors

In comparison to PD(ICD)− patients, PD gamblers have shown great decreases in post-synaptic dopamine receptors availabilities (as reflected by ^11^C-raclopride PET) in the ventral striatum [49]. By using (S)-N-(1-Ethyl-2-pyrrolidinyl)methyl)-5-bromo-2-^11^C-methoxy-3-methoxybenzamide (^11^C-FLB-457) PET (specific for extrastriatal post-synaptic dopamine D2/D3 receptors) PD patients who developed pathological gambling following treatment with DAg showed reduced dopamine receptor availabilities in the midbrain (tectum, cerebral aqueduct, tegmentum, and basis pedunculi), as compared to treated PD(ICD)− patients [50]. The degree of midbrain ^11^C-FLB-457 binding correlated with impulsivity clinical scores and was significantly higher in the anterior cingulate cortex of PD pathological gamblers [50].

PD patients with either single or multiple ICDs release significantly greater amounts of synaptic dopamine in the ventral striatum as compared to PD(ICD)− individuals, in response to visual reward cues [51, 52]. Of interest, as reflected by changes in ^11^C-raclopride binding in the ventral striatum, PD patients with single ICD were no different from the patients who had multiple ICDs, while from a clinical point of view, PD patients with multiple ICDs were more depressed and had higher levels of impulsive sensation seeking as compared to those with single ICD as well as to PD patients without an ICD history [52].

By using ^11^C-raclopride PET, pathological gamblers with PD have shown decreased ventral striatum binding that correlated with greater temporal discounting preferences i.e. tendency to discount rewards that move away from now [53]. Temporal discounting also correlated with lower ventral striatal dopamine release in response to high-reward magnitude [53]. In the same report, temporal discounting correlated with greater dopaminergic terminal function in the left caudate and anterior putamen (assessed with ^18^F-dopa PET) [53]. In another report [11C](+)-4-N-Propyl-,3,4a,5,6,10b-hexahydro-2H-naphth[1,2-b][1,4]-oxazin-9-ol ([^11^C](+)-PHNO) PET binding was lower in the ventral striatum of the PD(ICD)+ patients and in negative correlation with clinical measures of ICD severity [54]. Interestingly, [^11^C](+)-PHNO binding was increased in the dorsal striatum across all PD patients in comparison to healthy controls [54]. It is likely that the above results reflect higher dopamine tone in the ventral striatum of PD(ICD)+ patients.

In a more recent study, PD(ICD)+ patients had reduced post-synaptic dopamine receptor densities [as reflected by (S)-N-((1-Allyl-2-pyrrolidinyl)methyl)-5-(3-^18^F-fluoropropyl)-2,3-dimethoxybenzamide (^18^F-fallypride) PET] in the ventral striatum and the putamen, while self-reported severity of ICD positively correlated with ^18^F-Fallypride binding in the midbrain [55••]. However, midbrain ^18^F-fallypride binding was no different between PD(ICD)+ and PD(ICD)− groups [55••]. The authors concluded that ventral midbrain dopamine projections throughout the nigrostriatal and mesolimbic networks are relatively preserved in PD-related ICD, and that this may partly account for the differential response PD(ICD)+ patients have to various DAg. It is likely that compulsive behaviours in PD are associated with reduced ventral and dorsal striatal post-synaptic dopamine receptor expression.

### Targeting the Pre-synaptic Membrane

Pre-synaptic markers of the dopaminergic system synapse are believed to be relatively unaffected by chronic exposure to dopaminergic agents. Among these, the dopamine transporter (DAT) is considered a relatively robust target to study the integrity of dopaminergic terminals. Nevertheless, given that DAT is highly expressed within the dorsal striatum, DAT-specific imaging in PD has been most commonly used in PD diagnostics and less in studying the non-motor aspects of PD.

By using DAT-specific N-(3-^18^F-Fluoropropyl)-2β-carbomethoxy-3β-(4-iodophenyl)nortropane (^18^F-FP-CIT) PET, PD(ICD)+ patients have shown significantly higher DAT density in the dorsal posterior cingulate and insular cortices as compared to PD(ICD)− [56]. However, in the ventral striatum and ventral pallidum DAT-specific binding appears reduced in PD(ICD)+ subjects [56, 57]. Relative to PD(ICD)− patients, PD patients who later developed ICD had lower baseline DAT densities in the ventral striatum, anterior-dorsal striatum and posterior putamen before ICD symptom onset, alongside higher depression scores [58]. In the same report, ICD severity correlated negatively with baseline (pre-ICD) DAT in the ventral and anterior dorsal striatum [58]. Taken together, it could be argued that lower DAT density in the ventral striatum is likely to precede the future development of PD-related ICD. In a similar context, Smith and colleagues evaluated the risk of developing ICD in a large cohort of de novo PD patients. In a longitudinal design, they used multiple DAT-specific SPECT imaging and showed that lower putaminal and total striatal DAT densities at baseline precede the future development of ICD [59]. Thus, in PD, lower striatal DAT density and in particular the ventral portion appears to be an additional risk factor for the future development of ICD.

### Multimodal Imaging

More recently, an interesting multimodal imaging study combined fMRI with PET data from a large group of PD patients. A negative correlation was found between dopamine synthesis capacity (as reflected by ^18^F-dopa PET) in nucleus accumbens and impulsivity severity (as reflected by appropriate ICD rating scores), suggesting that nucleus accumbens, which is functionally connected to the rostral anterior cingulate cortex, is significantly affected in PD-related ICD [60, 61•]. From this cohort, patients with more severe ICD had reduced functional connectivity between nucleus accumbens and rostral anterior cingulate cortex. Further to that, cortical thickness and severity of ICD were positively correlated in the subgenual rostral anterior cingulate cortex [61•].

## Conclusions

In summary of the results discussed above, PD-related ICD occurs due to structural as well as molecular alterations. Neuroimaging studies suggest that ICD develops in PD on a pre-existing dysfunctional dopaminergic reward system. However, it is not quite clear whether the above pathophysiology extends to other non-dopaminergic systems (e.g. serotonin).

It appears that ICD develops in some PD individuals from interactions between prescribed dopaminergic drugs and pre-existing structural and functional alterations. Increased dopamine release in the ventral striatum and possibly reduced expression of post-synaptic dopamine receptors form the background mechanisms, on which DAg act to trigger ICD. Constant stimulation by DRT agents may therefore negatively affect the relatively preserved ventral striatum by exacerbating the lack of control within the reward system. In addition, dysfunction in the amygdala and abnormal ventromedial subthalamic nucleus stimulation may contribute to the above dynamics and exacerbate ICD. Future neuroimaging studies should focus on clarifying the complex interactions of all involved neuronal networks and improve the current strategy for managing PD-related ICD.

## References

[1] Joutsa J, Martikainen K, Vahlberg T, Voon V, Kaasinen V (2012). Impulse control disorders and depression in Finnish patients with Parkinson's disease. Parkinsonism Relat Disord.

[2] Weintraub D, Nirenberg MJ (2013). Impulse control and related disorders in Parkinson's disease. Neurodegener Dis.

[3] Voon V, Napier TC, Frank MJ, Sgambato-Faure V, Grace AA, Rodriguez-Oroz M, Obeso J, Bezard E, Fernagut PO (2017). Impulse control disorders and levodopa-induced dyskinesias in Parkinson's disease: an update. Lancet Neurol.

[4] Ramirez-Zamora A, Gee L, Boyd J, Biller J (2016). Treatment of impulse control disorders in Parkinson's disease: practical considerations and future directions. Expert Rev Neurother.

[5] National Institute for Health and Care Excellence [NICE], 2017; https://www.nice.org.uk/guidance/ng71/chapter/Recommendations#managing-and-monitoring-impulse-control-disorders-as-an-adverse-effect-of-dopaminergic-therapy

[6] Sturm V, Lenartz D, Koulousakis A, Treuer H, Herholz K, Klein JC, Klosterkötter J (2003). The nucleus accumbens: a target for deep brain stimulation in obsessive-compulsive- and anxiety-disorders. J Chem Neuroanat.

[7] Franzini A, Messina G, Gambini O, Muffatti R, Scarone S, Cordella R, Broggi G (2010). Deep-brain stimulation of the nucleus accumbens in obsessive compulsive disorder: clinical, surgical and electrophysiological considerations in two consecutive patients. Neurol Sci.

[8] Erga AH, Alves G, Larsen JP, Tysnes OB, Pedersen KF (2017). Impulsive and compulsive behaviors in Parkinson's disease: the Norwegian ParkWest study. J Park Dis.

[9] Rodríguez-Violante M, González-Latapi P, Cervantes-Arriaga A, Camacho-Ordoñez A, Weintraub D (2014). Impulse control and related disorders in Mexican Parkinson's disease patients. Parkinsonism Relat Disord.

[10] Antonini A, Barone P, Bonuccelli U, Annoni K, Asgharnejad M, Stanzione P (2017). ICARUS study: prevalence and clinical features of impulse control disorders in Parkinson's disease. J Neurol Neurosurg Psychiatry.

[11] Sharma A, Goyal V, Behari M, Srivastva A, Shukla G, Vibha D (2015). Impulse control disorders and related behaviours (ICD-RBs) in Parkinson's disease patients: assessment using “questionnaire for impulsive-compulsive disorders in Parkinson's disease” (QUIP). Ann Indian Acad Neurol.

[12] Napier TC, Corvol JC, Grace AA, Roitman JD, Rowe J, Voon V, Strafella AP (2015). Linking neuroscience with modern concepts of impulse control disorders in Parkinson's disease. Mov Disord.

[13] Tippmann-Peikert M, Park JG, Boeve BF, Shepard JW, Silber MH (2007). Pathologic gambling in patients with restless legs syndrome treated with dopaminergic agonists. Neurology.

[14] Evans AH, Stegeman JR (2009). Punding in patients on dopamine agonists for restless leg syndrome. Mov Disord.

[15] Fan W, Ding H, Ma J, Chan P (2009). Impulse control disorders in Parkinson's disease in a Chinese population. Neurosci Lett.

[16] Weintraub D, Koester J, Potenza MN, Siderowf AD, Stacy M, Voon V, Whetteckey J, Wunderlich GR, Lang AE (2010). Impulse control disorders in Parkinson disease: a cross-sectional study of 3090 patients. Arch Neurol.

[17] Perez-Lloret S, Rey MV, Fabre N, Ory F, Spampinato U, Brefel-Courbon C, Montastruc JL, Rascol O (2012). Prevalence and pharmacological factors associated with impulse-control disorder symptoms in patients with Parkinson disease. Clin Neuropharmacol.

[18] Ambermoon P, Carter A, Hall WD, Dissanayaka NN, O'Sullivan JD (2011). Impulse control disorders in patients with Parkinson's disease receiving dopamine replacement therapy: evidence and implications for the addictions field. Addiction.

[19] Voon V, Mehta AR, Hallett M (2011). Impulse control disorders in Parkinson's disease: recent advances. Curr Opin Neurol.

[20] Lorains FK, Cowlishaw S, Thomas SA (2011). Prevalence of comorbid disorders in problem and pathological gambling: systematic review and meta-analysis of population surveys. Addiction.

[21] Coleman E, Raymond N, McBean A (2003). Assessment and treatment of compulsive sexual behavior. Minn Med.

[22] Lejoyeux M, Weinstein A (2010). Compulsive buying. Am J Drug Alcohol Abuse.

[23] Lejoyeux M, Lehert P (2011). Alcohol-use disorders and depression: results from individual patient data meta-analysis of the acamprosate-controlled studies. Alcohol Alcohol.

[24] Strafella AP (2019). Mesolimbic dopamine and anterior cingulate cortex connectivity changes lead to impulsive behaviour in Parkinson's disease. Brain.

[25] Robbins TW, Everitt BJ (1996). Neurobehavioural mechanisms of reward and motivation. Curr Opin Neurobiol.

[26] Schultz W, Dayan P, Montague PR (1997). A neural substrate of prediction and reward. Science.

[27] Park YS, Sammartino F, Young NA, Corrigan J, Krishna V, Rezai AR (2019). Anatomical review of the ventral capsule/ventral striatum and the nucleus accumbens to guide target selection for deep brain stimulation for obsessive-compulsive disorder. World Neurosurg.

[28] Fudge JL, Haber SN (2002). Defining the caudal ventral striatum in primates: cellular and histochemical features. J Neurosci.

[29] Heimer L (2003). A new anatomical framework for neuropsychiatric disorders and drug abuse. Am J Psychiatry.

[30] Rao H, Mamikonyan E, Detre JA, Siderowf AD, Stern MB, Potenza MN, Weintraub D (2010). Decreased ventral striatal activity with impulse control disorders in Parkinson's disease. Mov Disord.

[31] Petersen K, Van Wouwe N, Stark A, Lin YC, Kang H, Trujillo-Diaz P (2018). Ventral striatal network connectivity reflects reward learning and behavior in patients with Parkinson’s disease. Hum Brain Mapp.

[32] Carriere N, Lopes R, Defebvre L, Delmaire C, Dujardin K (2015). Impaired corticostriatal connectivity in impulse control disorders in Parkinson disease. Neurology.

[33] Tessitore A, Santangelo G, De Micco R, Giordano A, Raimo S, Amboni M (2017). Resting-state brain networks in patients with Parkinson's disease and impulse control disorders. Cortex.

[34] Markovic V, Agosta F, Canu E, Inuggi A, Petrovic I, Stankovic I, Imperiale F, Stojkovic T, Kostic VS, Filippi M (2017). Role of habenula and amygdala dysfunction in Parkinson disease patients with punding. Neurology.

[35] Filip P, Linhartová P, Hlavatá P, Šumec R, Baláž M, Bareš M, Kašpárek T (2018). Disruption of multiple distinctive neural networks associated with impulse control disorder in Parkinson's disease. Front Hum Neurosci.

[36] Pellicano C, Niccolini F, Wu K, O'Sullivan SS, Lawrence AD, Lees AJ (2015). Morphometric changes in the reward system of Parkinson's disease patients with impulse control disorders. J Neurol.

[37] Tessitore A, Santangelo G, De Micco R, Vitale C, Giordano A, Raimo S (2016). Cortical thickness changes in patients with Parkinson’s disease and impulse control disorders. Parkinsonism Relat Disord.

[38] Ricciardi L, Lambert C, De Micco R, Morgante F, Edwards M (2018). Can we predict development of impulsive-compulsive behaviours in Parkinson's disease?. J Neurol Neurosurg Psychiatry.

[39] Imperiale F, Agosta F, Canu E, Markovic V, Inuggi A, Jecmenica-Lukic M, Tomic A, Copetti M, Basaia S, Kostic VS, Filippi M (2018). Brain structural and functional signatures of impulsive-compulsive behaviours in Parkinson's disease. Mol Psychiatry.

[40] Biundo R, Weis L, Facchini S, Formento-Dojot P, Vallelunga A, Pilleri M, Weintraub D, Antonini A (2015). Patterns of cortical thickness associated with impulse control disorders in Parkinson's disease. Mov Disord.

[41] Canu E, Agosta F, Markovic V, Petrovic I, Stankovic I, Imperiale F, Stojkovic T, Copetti M, Kostic VS, Filippi M (2017). White matter tract alterations in Parkinson's disease patients with punding. Parkinsonism Relat Disord.

[42] Mojtahed Zadeh M, Ashraf-Ganjouei A, Ghazi Sherbaf F, Haghshomar M, Aarabi MH (2018). White matter tract alterations in drug-Naïve Parkinson's disease patients with impulse control disorders. Front Neurol.

[43] Yoo HB, Lee JY, Lee JS, Kang H, Kim YK, Song IC, Lee DS, Jeon BS (2015). Whole-brain diffusion-tensor changes in parkinsonian patients with impulse control disorders. J Clin Neurol.

[44] Thiel A, Hilker R, Kessler J, Habedank B, Herholz K, Heiss WD (2003). Activation of basal ganglia loops in idiopathic Parkinson's disease: a PET study. J Neural Transm (Vienna).

[45] van Eimeren T, Pellecchia G, Cilia R, Ballanger B, Steeves TD, Houle S (2010). Drug-induced deactivation of inhibitory networks predicts pathological gambling in PD. Neurology.

[46] Antonelli F, Ko JH, Miyasaki J, Lang AE, Houle S, Valzania F, Ray NJ, Strafella AP (2014). Dopamine-agonists and impulsivity in Parkinson's disease: impulsive choices vs. impulsive actions. Hum Brain Mapp.

[47] Tahmasian M, Rochhausen L, Maier F, Williamson KL, Drzezga A, Timmermann L (2015). Impulsivity is associated with increased metabolism in the Fronto-insular network in Parkinson's disease. Front Behav Neurosci.

[48] Verger A, Klesse E, Chawki MB, Witjas T, Azulay JP, Eusebio A, Guedj E (2018). Brain PET substrate of impulse control disorders in Parkinson's disease: a metabolic connectivity study. Hum Brain Mapp.

[49] Steeves TD, Miyasaki J, Zurowski M, Lang AE, Pellecchia G, Van Eimeren T (2009). Increased striatal dopamine release in Parkinsonian patients with pathological gambling: a [11C] raclopride PET study. Brain.

[50] Ray NJ, Miyasaki JM, Zurowski M, Ko JH, Cho SS, Pellecchia G, Antonelli F, Houle S, Lang AE, Strafella AP (2012). Extrastriatal dopaminergic abnormalities of DA homeostasis in Parkinson's patients with medication-induced pathological gambling: a [11C] FLB-457 and PET study. Neurobiol Dis.

[51] O'Sullivan SS, Wu K, Politis M, Lawrence AD, Evans AH, Bose SK, Djamshidian A, Lees AJ, Piccini P (2011). Cue-induced striatal dopamine release in Parkinson's disease-associated impulsive-compulsive behaviours. Brain.

[52] Wu K, Politis M, O'Sullivan SS, Lawrence AD, Warsi S, Bose S (2015). Single versus multiple impulse control disorders in Parkinson's disease: an ^11^C-raclopride positron emission tomography study of reward cue-evoked striatal dopamine release. J Neurol.

[53] Joutsa J, Voon V, Johansson J, Niemelä S, Bergman J, Kaasinen V (2015). Dopaminergic function and intertemporal choice. Transl Psychiatry.

[54] Payer DE, Guttman M, Kish SJ, Tong J, Strafella A, Zack M, Adams JR, Rusjan P, Houle S, Furukawa Y, Wilson AA, Boileau I (2015). [^11^C]-(+)-PHNO PET imaging of dopamine D(2/3) receptors in Parkinson's disease with impulse control disorders. Mov Disord.

[55] Stark AJ, Smith CT, Lin YC, Petersen KJ, Trujillo P, van Wouwe NC (2018). Nigrostriatal and mesolimbic D2/3 receptor expression in Parkinson's disease patients with compulsive reward-driven behaviors. J Neurosci.

[56] Lee JY, Seo SH, Kim YK, Yoo HB, Kim YE, Song IC, Lee JS, Jeon BS (2014). Extrastriatal dopaminergic changes in Parkinson's disease patients with impulse control disorders. J Neurol Neurosurg Psychiatry.

[57] Voon V, Rizos A, Chakravartty R, Mulholland N, Robinson S, Howell NA, Harrison N, Vivian G, Ray Chaudhuri K (2014). Impulse control disorders in Parkinson's disease: decreased striatal dopamine transporter levels. J Neurol Neurosurg Psychiatry.

[58] Vriend C, Nordbeck AH, Booij J, van der Werf YD, Pattij T, Voorn P, Raijmakers P, Foncke EM, van de Giessen E, Berendse HW, van den Heuvel OA (2014). Reduced dopamine transporter binding predates impulse control disorders in Parkinson's disease. Mov Disord.

[59] Smith KM, Xie SX, Weintraub D (2016). Incident impulse control disorder symptoms and dopamine transporter imaging in Parkinson disease. J Neurol Neurosurg Psychiatry.

[60] Weintraub D, Mamikonyan E, Papay K, Shea JA, Xie SX, Siderowf A (2012). Questionnaire for impulsive-compulsive disorders in Parkinson's disease-rating scale. Mov Disord.

[61] Hammes J, Theis H, Giehl K, Hoenig MC, Greuel A, Tittgemeyer M, Timmermann L, Fink GR, Drzezga A, Eggers C, van Eimeren T (2019). Dopamine metabolism of the nucleus accumbens and fronto-striatal connectivity modulate impulse control. Brain.

